# Dr. Johnson and the British and Foreign Medical Review

**Published:** 1838-07-01

**Authors:** 


### DR. JOHNSON

AND

### THE BRITISH AND FOREIGN MEDICAL REVIEW.

---

To Drs. Forbes and Connolly,

*Editors of the British and Foreign Medical Review.*

Gentlemen and Chers Confreres!

I cannot sufficiently express the obligations I am under for your highly liberal, impartial, and honorable conduct in the review of my late work, in the last number of your journal—a journal which is acknowledged, even in the wigwams of America, to be the "*first in the world.*" I confess that I am not without some compunctious visitings of conscience, on this occasion, when I reflect on the very *different* manner in which I treated *your* works when they came under my critical notice a few years ago. Ingratitude is a crime which few men like to own—but I am compelled to the admission of it, by the striking contrast between the tone and manner adopted in the two journals towards their respective Editors. Many an illnatured Reviewer would have seized such an opportunity as you have had, to retaliate upon me the unfriendly, illiberal, and unjust criticisms which I once bestowed on the "Treatise on Insanity," and the "Translation of Laennec." But you, gentlemen, are far superior to such considerations. You scorn to take advantage of a Review to imitate my bad example of lavishing general and indiscriminate abuse on books, without quoting a single passage to substantiate the charges! No, you have returned good for evil—eulogies for censures—and thus punished me nobly, but effectually, for my manifold acts of injustice towards you! The ways in which you have dealt so kindly with me are numerous and judicious. You are aware that the public look much more to the *matter* than to the *manner* of a book; and, conscious that the *former*, in mine, would not bear exposure to public view, you have, with equal charity and justice, kept it entirely out of sight—dwelling on the beauties of the style, with a delight which, I fear, your readers will perceive to be a mark of partiality to the author, not calculated to enhance the decision of the critic.

The ingenious mode which you have adopted to display the merits of my style, is equally original and *ingenuous*. Instead of pursuing the beaten path of selecting extracts to justify your criticisms, you have picked out one or two words from various paragraphs, and the paragraphs themselves from various pages, or even chapters of my work, and then, by twirling them round in your literary Kaleidoscope, you have exhibited them to the world as authentic specimens of the manner in which my book is composed*—or

---

* The following is one of the specimens of my style exhibited by my impartial Reviewers. "Health, Time, Poverty, Affluence, Fame, Power, Sceptic, Prince, Beauty, Literature, Science, Religion, Cholera, Hygiene, Community."

rather of the art with which you have contrived to decompose it—and all for the benefit of the author, the advancement of medical science, and the respectability of that profession, of which you have very properly constituted yourselves the arbiters and representatives.

To shew the public however that you do not always hold the scales of justice with a bandage on your eyes, and that you are not totally blind to the blemishes of your friend's book, you have lightly touched, with healing hand, on some of his failings. Thus you gently insinuate that the sole aim and object of my writings are to acquire pelf—in short, that every sentence is meant to convey this and this only—" TRY WARREN." Lack of work and lack of means might plead some excuse on my part; but fortunately the same could not be pleaded, if any *puffery* were exhibited by my Reviewers.

Most of your readers must have been highly gratified by seeing the following paragraph in every Newspaper and periodical in England :—" THE BRITISH AND FOREIGN MEDICAL REVIEW IS DECIDEDLY THE FIRST PERIODICAL IN THE WORLD."—" *American Library*." This advertisement is of course, inserted daily against your express commands. Coming from any other source, it would be considered very like a puff—and quite equal to—" TRY WARREN ;" but the intentional and literal meaning of it is neither more nor less than this :—" *try the Edinburgh or Medico-Chirurgical Quarterlies—don't think of the British and Foreign.*"

But now to be serious. The gravest charge against me is, that, by attractive headings, and other typical artifices, I have contrived to raise the hopes of my readers, only to disappoint their expectations. On this point, gentlemen, we have a small account to settle, and the balance of merit shall be quarterly struck in future between us.

The first department of your last Number, viz. " *Analytical* and Critical Reviews," consists of 215 pages. And how many pages of letter-press do the works reviewed contain? Only TEN THOUSAND SEVEN HUNDRED AND NINETY-FIVE PAGES ! ! Now the *reading alone* of this small modicum of matter in the original works, would require, at the rate of two minutes to the page (a rapid rate), a labour of four hours per diem for ninety days ; namely, for a quarter of a year ! This is putting entirely aside the arduous task of analyzing, criticising, reflecting on—and last, not least, of writing out the Reviews ! If the attention which is considered necessary to these last items of work, are actually paid, why then a corps of Reviewers, amounting to at least twenty, and working twelve hours a day, would hardly be sufficient for a single department of your Journal !* This little statistical calculation will throw some light on the noble " art and mystery" of reviewing ; but the most important consideration for the public is yet to come. How much of the materiel contained in the original works is transferred to the pages of your Journal for the good of your readers? Granting that every line in these analytical reviews contained nothing but the pure quintescence of the books reviewed, there would be about *one line* extracted, on an

---

* I purposely avoid the " Bibliographical Notices"—and the Foreign and domestic journals, because no estimate can possibly be made of the labour and time bestowed upon them—and because they are not professed to be " Analytical Reviews."

average, from each *page* of the original publications! But when we exhumate the few unconnected and unabridged quotations out of the mass of twaddle in which the egotism of the Reviewer has embedded them, we may fairly calculate that the shortest monosyllable in the English language would be equal to the nutritive matter extracted from each folio of the works analyzed! Thus homœopathy, in all its glory, is fused into the art and science of modern journalism, and the reader is treated with infinitessimal doses of knowledge, while the laughing juggler behind the scene assures him, ever and anon, that he is swallowing enormous allopathic potations of that invaluable commodity! In short, a more unblushing specimen of literary deception, critical quackery, and thimble-rig analysis, has never, perhaps, been exhibited, since the invention of printing!

Now, gentlemen, I do not maintain that, in all the "analytical and critical reviews," of your quarterly, the same exact relative proportion between the size of the volume reviewed and the length of the article in the Journal, is preserved. I admit that you exercise a sound discretion, on this point; while the general average remains nearly the same. I will adduce a striking example of your discrimination, from the article which has called forth this letter.

Thus out of nearly 300 pages of my work, not a single useful hint is extracted—not a single error corrected! You have carefully sifted the whole volume—and you have presented the chaff, and the chaff only, (criticisms on style) to your readers. One of the most substantial extracts, is the catalogue of naked words selected from different pages, which may be seen in the foot note a little farther back. There they stand, isolated, mute, and unintelligible as the granite blocks of the Druids' circle on Salisbury Plain, while, from a certain other work in the leash, which need not be named, but which is well known to be not less distinguished for the *originality* of its composition, than for the *genius* of its author, large quotations are drawn, with corresponding encomia—a procedure which marks, at once, the practical discernment of the reviewer, and the rigid impartiality of the critic.

And yet, gentlemen, there is nothing new under the sun—not even your own thimble-rig system of reviewing. It is now just twenty years since I started a Journal on diametrically opposite principles, viz. that of epitomizing rather than criticising the works reviewed—of selecting and concentrating the useful *matter* from the mass of *verbiage* in which it usually is enveloped—and appending practical comments where my experience enabled me to detect error or confirm truth. This plan brought about my ears a hornet's nest of three different classes of the community—the authors, the publishers, and the journalists. The authors declared that I trepanned their skulls and extracted their brains*—the publishers complained that I injured the sale of their books, since many practitioners preferred the analysis to the original—but the journalists were furious against me for introducing a system that imposed a heavy labour on the reviewer which, however beneficial to the reader, was highly injurious to the *trade* of journalism, by substituting laborious analysis for light and airy criticism. The system was

* The late Sir David Barry took counsel's opinion on the propriety of bringing an action against me for PIRACY!

therefore to be crushed, and a "learned Theban" of that day undertook to exterminate, at once, both editor and journal. For this purpose one of my works was selected for immolation, and, by means of misquotation, misrepresentation, *falsification*, and various other arts well known in the *trade*, the Reviewer contrived, with great ingenuity, to hold me up to the derision and contempt of the public, as a man totally ignorant of his profession, and, of course, incapacitated for editing a medical journal. With a consistency remarkable in great critics, the same book was reviewed by the same writer, and in the same journal, with strong encomiums—only one short year previously! But *then* the author was in a provincial town, and had not started a rival journal!*

To make assurance doubly sure, and not content with this savage assault on myself, he determined on sweeping the MEDICO-CHIRURGICAL REVIEW from the face of the earth. For this purpose he set to in good earnest, and reviewed his "TEN THOUSAND SEVEN HUNDRED AND NINETY-FIVE" pages of original authors, pounding and compressing their naked bones into his narrow sarcophagus—wisely calculating that such a quintescence—such a consommé of medical lore would supersede all other articles of the kind in the literary market. But Athenian ingratitude is not confined to the shores of Attica. The book-worms praised the learned labours of the Reviewer—the public permitted them to repose quietly in St. Paul's Church-yard. "Virtus laudatur et alget." The learned Theban threw up his brief in disgust, and the journal itself soon died of asphyxia, and vanished from the scene!

Whether I shall survive this second onslaught, can only be known to the FATES, whose inexorable shears may now, perhaps, be closing on my slender thread of existence, under the iron grasp of my marble-hearted executioners. But twenty years of editorial office might satisfy the ambition of any man; and, if gentlemen, you hold office as long, I venture to prophecy that, before half the twenty years have expired, you will reform your literary Kaleidoscope, filled with glittering baubles—your pedler's pack, crammed with samples of every thing, and substance of nothing—with fag-ends clipped from the tough yarns of Germany—the tinsel tissues of France—and the tawdry remnants of home manufactures—all tricked out, with ad captandum skill, for hawking through the provincial fairs, and astonishing the weak minds of the natives.

I am, Gentlemen,

With (of course) great respect,

Your most devoted humble servant,

*20th June*, 1838. JAMES JOHNSON.

---

* There is one remarkable coincidence between my Reviewers of 1818—and 1838. They both, and no doubt from the most disinterested motives, advise me to "write no more." I have not the smallest doubt of their entire *sincerity* in giving this advice.

